# Correction to ‘The human insulin receptor mRNA contains a functional internal ribosome entry segment’

**DOI:** 10.1093/nar/gkac063

**Published:** 2022-01-31

**Authors:** Keith A Spriggs, Laura C Cobbold, Simon H Ridley, Mark Coldwell, Andrew Bottley, Martin Bushell, Anne E Willis, Kenneth Siddle

**Affiliations:** University of Nottingham, School of Pharmacy, University Park, Nottingham NG7 2RD; University of Nottingham, School of Pharmacy, University Park, Nottingham NG7 2RD; University of Cambridge, Department of Clinical Biochemistry, Metabolic Research Laboratories, Institute of Metabolic Science, Addenbrooke's Hospital, Cambridge CB2 0QQ; School of Biological Sciences, University of Southampton, Boldrewood Campus, Bassett Crescent East, Southampton, SO16 7PX, UK; University of Nottingham, School of Pharmacy, University Park, Nottingham NG7 2RD; University of Nottingham, School of Pharmacy, University Park, Nottingham NG7 2RD; University of Nottingham, School of Pharmacy, University Park, Nottingham NG7 2RD; University of Cambridge, Department of Clinical Biochemistry, Metabolic Research Laboratories, Institute of Metabolic Science, Addenbrooke's Hospital, Cambridge CB2 0QQ

*Nucleic Acids Research*, Volume 37, Issue 17, 1 September 2009, Pages 5881–5893, https://doi.org/10.1093/nar/gkp623

The Editors were alerted in 2014 that some Electrophoretic Mobility Shift Assays (EMSAs) depicted in Fig4Bii showed unusual levels of similarity (panels 3a, 4b and 5d). The journal investigated the matter at the time and did not find conclusive evidence to support the allegations. The same allegations were brought to the Editors’ attention again in 2021.

Below is Figure 4Bii as originally published in 2009. Panel 2a shows signs of splicing. Bands 2-3 in panel 3a and bands 1-2 in panel 5d are unexpectedly similar. Bands 1-2 in panel 4b and bands 2-3 in panel 5d are unexpectedly similar.


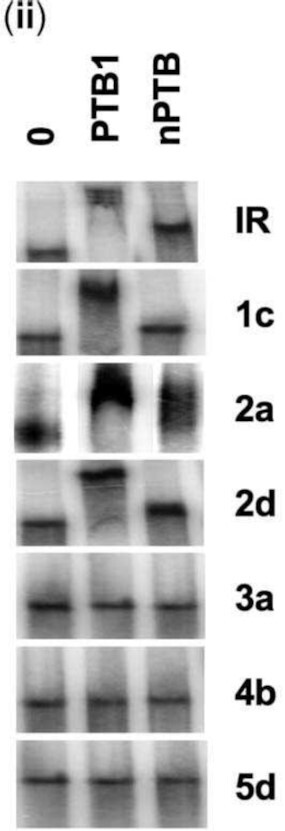


Below is the original EMSA used for panel 2a, the first three lanes were used. The original panel 2a was created without any splicing of individual lanes. However, the brightness/contrast of individual tracks was inappropriately adjusted in an attempt to standardise intensity, introducing signs of splicing.


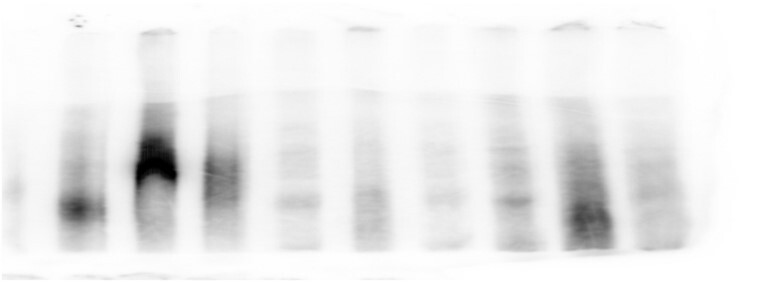


The corresponding authors acknowledge with regret errors in the preparation of panels 3a, 4b and 5d of Figure 4B (ii). Panels 3a, 4b and 5d were originally run in a single experiment and it appears that errors occurred during Figure assembly when lanes were incorrectly cut and thus accidentally duplicated. The image of the original EMSAs is no longer available but the authors provide below surviving images of replicate parallel experiments conducted around the same time in 2005/6. These images are of lesser quality and were therefore not used to construct the original figure.


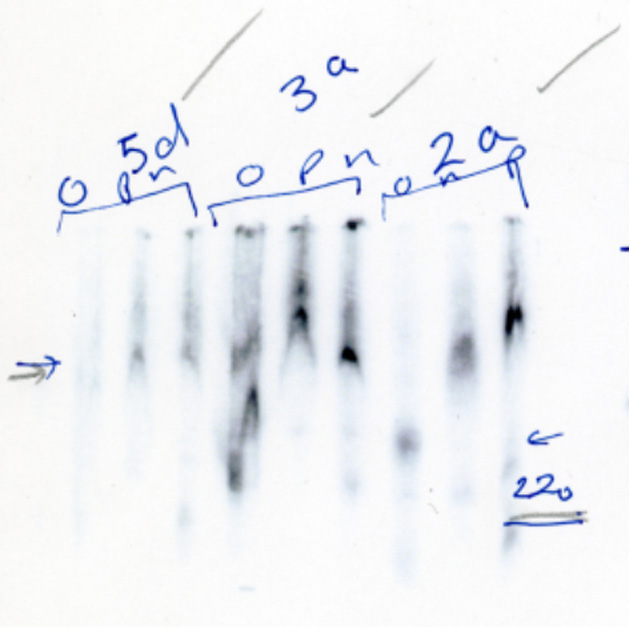



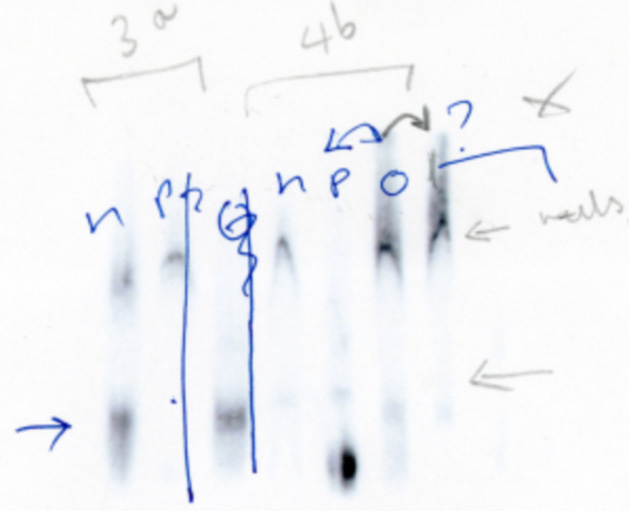


Using these images, the authors are providing a new Figure 4Bii.


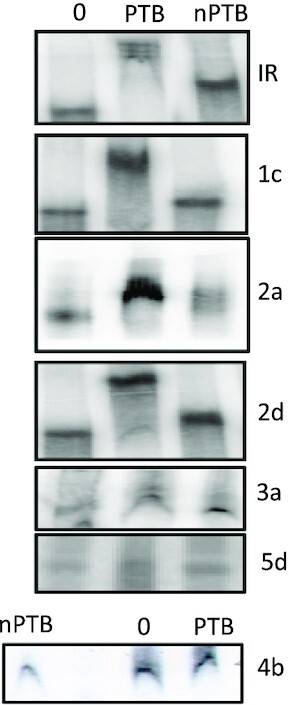


**New Figure 4Bii**. Electromobility shift assays were performed using radiolabelled fragments of IR-IRES RNA that had been incubated with 0.2 mg of PTB1 or nPTB. Products were separated on 5% TBE polyacrylamide gels. IR, 1c and 2d are from the original figure. Panel 2a is a new image of the same gel as in the original Figure. Panels 3a, 4b and 5d are from replicate parallel experiments and replace 3a, 4b and 5d of the original Figure.

